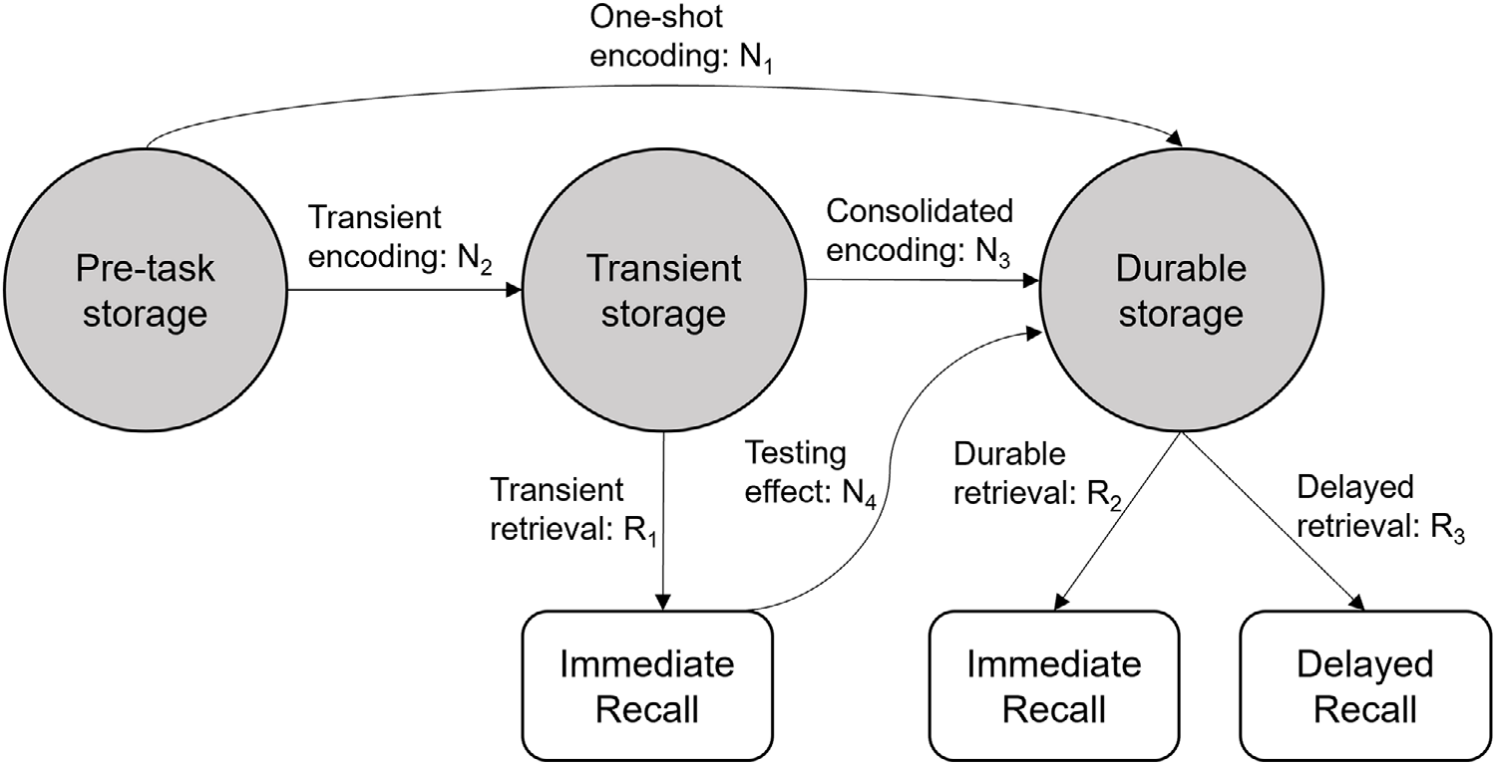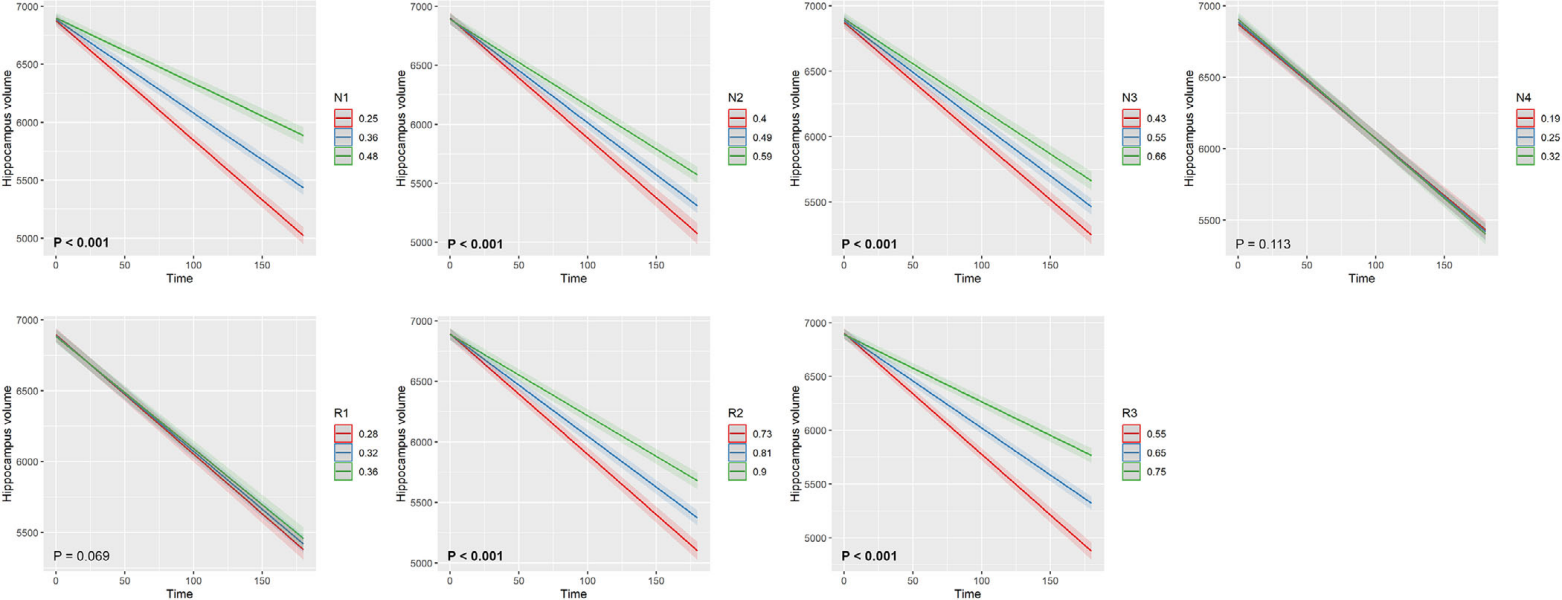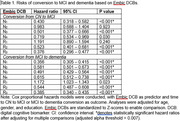# Utility of Embic digital cognitive biomarkers in predicting dementia conversion, brain volumetrics, and AD biomarkers in ADNI

**DOI:** 10.1002/alz.090088

**Published:** 2025-01-03

**Authors:** Michelle H. Chen, William T. Hu

**Affiliations:** ^1^ Rutgers Institute for Health, Health Care Policy, and Aging Research, New Brunswick, NJ USA; ^2^ Rutgers Robert Wood Johnson Medical School, New Brunswick, NJ USA; ^3^ Rutgers‐Robert Wood Johnson Medical School, New Brunswick, NJ USA

## Abstract

**Background:**

Early identification of preclinical Alzheimer’s disease (AD) is key to timely interventions. However, existing neuropsychological test scores are not sensitive to subtle cognitive decline during preclinical AD. There is a need to develop cognitive measures that are more sensitive to early stages of decline. Embic digital cognitive biomarkers (DCBs) apply hierarchical Bayesian cognitive processing model to item‐level data of word list memory measures and attempt to delineate latent processes of memory encoding and retrieval, which may be more sensitive to subtle cognitive decline. The current study examined the utility of Embic DCBs in predicting dementia conversion and changes in brain volumetrics, as well as the relationship between AD biomarkers and Embic DCB trajectories.

**Method:**

A total of 2,425 participants (596 were cognitively normal [CN] at baseline, 874 had mild cognitive impairment [MCI] at baseline, and 298 had dementia at baseline) in the Alzheimer’s Disease Neuroimaging Initiative (ADNI) with at least 3 visits were included in the current study. Cox proportional hazards models were conducted to determine conversion from CN to MCI and from MCI to dementia based on Embic DCB performance. Linear mixed effects models were performed to examine cross‐sectional and longitudinal associations between Embic DCBs and volumes of brain regions involved in memory, as well as Embic DCB trajectories based on cerebrospinal fluid (CSF)‐based AD biomarker profile.

**Result:**

Higher encoding and retrieval performances based on Embic DCBs were associated with increased risks of conversion from CN to MCI and from MCI to dementia. Specifically, measures related to encoding to and retrieval from long‐term “durable storage” were most predictive of conversion. Higher Embic DCB scores were associated with greater hippocampus and entorhinal cortex volumes at baseline, and those with lower Embic DCB scores exhibited greater decline in hippocampus and entorhinal cortex volumes over time. Participants with positive CSF‐based AD biomarkers showed steeper decline in Embic DCBs compared to those who were AD biomarker negative.

**Conclusion:**

The current study provided initial validation of Embic DCBs in predicting MCI/dementia conversion, brain volumetrics, and AD biomarkers in the ADNI sample. Future studies should examine the utility of Embic DCBs in predicting early stages of cognitive decline.